# Distinct DDX DEAD-box RNA helicases cooperate to modulate the HIV-1 Rev and Tat function

**DOI:** 10.1186/1742-4690-10-S1-O18

**Published:** 2013-09-19

**Authors:** Yasuo Ariumi, Mariko Yasuda-Inoue, Misao Kuroki

**Affiliations:** 1 Center for AIDS Research, Kumamoto University, Kumamoto, Japan

## Background

RNA helicase plays an important role in host mRNA and viral mRNA transcription, transport, and translation. Many viruses utilize RNA helicases in their life cycle, while human immunodeficiency virus type 1 (HIV-1) does not encode an RNA helicase. Thus, host RNA helicase has been involved in HIV-1 replication. Indeed, DDX1 and DDX3 DEAD-box RNA helicases are known to be required for efficient HIV-1 Rev-dependent RNA export. However, it remains unclear whether distinct DDX RNA helicases cross-talk and cooperate to modulate the HIV-1 Rev and Tat function.

## Materials and methods

To investigate the potential role of distinct DDX DEAD-box RNA helicases in HIV-1 Rev or Tat function, we used the Rev-dependent luciferase-based reporter plasmid pDM628 or HIV-1-LTR-Luc reporter plasmid. 293FT cells were cotransfected with several HA-tagged DDX, including DDX1, DDX3, DDX5, DDX6, DDX17, DDX21, DDX56, pDM628 or pHIV-1-Luc, and/or HIV-1 Rev or Tat expression plasmid. We also examined immunofluorescence and immunoprecipitation analysis to analyze the interaction of DDX with Rev or Tat protein.

## Results

In this study, we noticed that distinct DDX RNA helicases, including DDX1, DDX3, DDX5, DDX17, DDX21, DDX56, except DDX6, bound to the Rev protein and they colocalized with Rev in nucleolus or nucleus. In this context, these DEAD-box RNA helicases except DDX6 markedly enhanced the HIV-1 Rev-dependent RNA export. Furthermore, DDX3 interacted with DDX5 and synergistically enhanced the Rev function. As well, combination of other distinct DDX RNA helicases cooperated to stimulate the Rev function. On the other hand, DDX3 also interacted with HIV-1 Tat and modulated Tat-induced transcription. Indeed, the ATPase-dependent RNA helicase activity of DDX3 seemed to be important for both Rev and Tat function. In contrast, other nuclear and nucleolar DDX RNA helicases, including DDX5, DDX17, DDX21, and DDX56, could interact with HIV-1 Tat, while these DEAD-box RNA helicases did not coactivate the Tat-induced transcription.

## Conclusion

Altogether, these results suggest that distinct DDX DEAD-box RNA helicases cooperate to modulate the HIV-1 Rev and Tat function.

